# Artificial intelligence classification model for macular degeneration images: a robust optimization framework for residual neural networks

**DOI:** 10.1186/s12859-021-04085-9

**Published:** 2021-11-08

**Authors:** Wen-Hsien Ho, Tian-Hsiang Huang, Po-Yuan Yang, Jyh-Horng Chou, Hong-Siang Huang, Li-Chung Chi, Fu-I Chou, Jinn-Tsong Tsai

**Affiliations:** 1 Department of Healthcare Administration and Medical Informatics, Kaohsiung Medical University, No. 100, Shin-Chuan 1st Road, Kaohsiung, 807 Taiwan; 2 Department of Medical Research, Kaohsiung Medical University Hospital, No. 100, Shin-Chuan 1st Road, Kaohsiung, 807 Taiwan; 3 Center for Big Data Research, Kaohsiung Medical University, No. 100, Shin-Chuan 1st Road, Kaohsiung, 807 Taiwan; 4 Department of Information Engineering and Computer Science, Feng Chia University, No. 100, Wenhwa Road, Taichung, 407 Taiwan; 5 Department of Mechanical Engineering, National Chung-Hsing University, No. 145, Xingda Road, Taichung, 402 Taiwan; 6 Department of Electrical Engineering, National Kaohsiung University of Science and Technology, No. 415, Chien-Kung Road, Kaohsiung, 807 Taiwan; 7 Department of Ophthalmology, Kaohsiung Medical University Hospital, No. 100, Shin-Chuan 1st Road, Kaohsiung, 807 Taiwan; 8 Department of Ophthalmology, Kaohsiung Municipal SiaoGang Hospital, No. 482, Shanming Road, Kaohsiung, 812 Taiwan; 9 Department of Computer Science, National Pingtung University, No. 4-18, Min-Sheng Road, Pingtung, 900 Taiwan

**Keywords:** Residual Neural Network, Uniform experimental design, Hyperparameter optimization, Macular degeneration classification

## Abstract

**Background:**

The prevalence of chronic disease is growing in aging societies, and artificial-intelligence–assisted interpretation of macular degeneration images is a topic that merits research. This study proposes a residual neural network (ResNet) model constructed using uniform design. The ResNet model is an artificial intelligence model that classifies macular degeneration images and can assist medical professionals in related tests and classification tasks, enhance confidence in making diagnoses, and reassure patients. However, the various hyperparameters in a ResNet lead to the problem of hyperparameter optimization in the model. This study employed uniform design—a systematic, scientific experimental design—to optimize the hyperparameters of the ResNet and establish a ResNet with optimal robustness.

**Results:**

An open dataset of macular degeneration images (https://data.mendeley.com/datasets/rscbjbr9sj/3) was divided into training, validation, and test datasets. According to accuracy, false negative rate, and signal-to-noise ratio, this study used uniform design to determine the optimal combination of ResNet hyperparameters. The ResNet model was tested and the results compared with results obtained in a previous study using the same dataset. The ResNet model achieved higher optimal accuracy (0.9907), higher mean accuracy (0.9848), and a lower mean false negative rate (0.015) than did the model previously reported. The optimal ResNet hyperparameter combination identified using the uniform design method exhibited excellent performance.

**Conclusion:**

The high stability of the ResNet model established using uniform design is attributable to the study’s strict focus on achieving both high accuracy and low standard deviation. This study optimized the hyperparameters of the ResNet model by using uniform design because the design features uniform distribution of experimental points and facilitates effective determination of the representative parameter combination, reducing the time required for parameter design and fulfilling the requirements of a systematic parameter design process.

## Background

The macula is located at the center of the retina and is responsible for vision and color identification. Its full name, macula lutea, originates from its dark-yellow color (visible under an ophthalmoscope), which is attributable to its high lutein content. Macular degeneration comprises two forms, dry and wet. Choroidal neovascularization (CNV) and diabetic macular edeman (DME) are wet forms, whereas drusen is the dry form. Only 10% of patients with macular degeneration are diagnosed as having a wet form. Because the deterioration of vision is often mistaken as a sign of presbyopia, the symptoms are easily overlooked, which can lead to serious results such as vision loss [1, 2].

The diagnosis of retinal abnormalities requires an examination of the retina by a trained medical professional and retinal image data obtained through optical coherence tomography (OCT). However, determining the form of macular degeneration through medical imaging is a time- and labor-consuming process. Ophthalmologists receive professional training and engage in repeated inspections and discussions. In remote areas or areas with insufficient medical resources, medical technologists are the only frontline medical professionals, and they may not have the diagnostic capacity or confidence to make such a diagnosis. Accordingly, patients usually wait for weeks for the diagnostic result, which delays treatment and involves an enormous amount of labor and social resources [3, 4]. Artificial intelligence (AI) exhibits potential in quick, automatic classification of medical images, acceleration of diagnoses, and reduction of labor [5]. Appropriate applications of AI can greatly accelerate the examination of macular diseases and reduce the costs involved.

Various applications of AI in the diagnosis of eye diseases have been researched. For example, Sambaturu et al. [6] employed a convolutional neural network (CNN) to automatically delineate exudate and hemorrhage in fundus images in DME. In the following year, Gleryz and Ulusoy [7] used a CNN to segment retinal vessels and extract relevant vessel features (e.g., tortuosity, width, and length) for the diagnosis, treatment, and screening of diabetes and vascular diseases such as hypertension. The present study used a residual neural network (ResNet) for modeling, with the aim of distinguishing between macular diseases of various types.

Despite increasing applications of AI in the medical domain, scholars and researchers have predominantly focused their discussion and analysis on the accuracy of models but failed to consider model stability. Accordingly, with consideration of both model accuracy and stability, the present study aimed to construct an AI model with high accuracy and a stable recognition rate. It employed a systematic uniform design method to identify the optimal hyperparameter combination and thus determine the AI model optimal for relieving the diagnostic burden on medical personnel, reassuring patients, and maximizing patient satisfaction.

The uniform experimental design (UED) method developed by Wang and Fang [8–10] uses space filling designs to construct a set of experimental points uniformly scattered in a continuous design parameter space. Because it only considers uniform dispersion and not comparable orderliness, UED minimizes the number of experiments needed to acquire all available information. Therefore, the UED is very suitable for solving problems involving multiple factors with multiple levels.

To verify the AI retinal-disease classification model established using a ResNet, the model was compared with CNN, an artificial neural network proposed by Najeeb et al. [11].

## Methods

For the diagnosis of macular degeneration, a ResNet model was constructed with hyperparameters optimized using uniform design. With a focus on model accuracy and low standard deviation, this study proposed a method for establishing an accurate, low-standard deviation AI classification model and applied the model in prediction based on actual medical images to test its performance. The following describes the data collection process and design focus of the proposed method, focusing on the experimental design used to optimize the classification model.

**Fig. 1:**
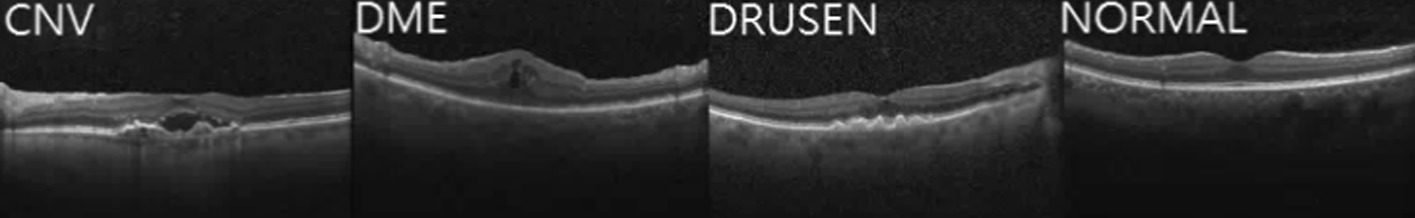
OCT image data [9]

The modeling was conducted using an OCT image dataset published by Kermany et al. [12], which comprised four categories of data: CNV, DME, drusen, and normal data. Figure 1 presents data for the categories, and Table 1 illustrates the numbers of data points in each data category. A total of 83,484 images were used for modeling, with 37,205 CNV, 11,348 DME, 8616 drusen, and 26,315 normal images; 968 images were employed for model testing, with 242 images for each of the categories.

**Table 1:** Number of images for macular degeneration modeling and testing

Type of macular degeneration	Number of modeling data points	Number of test data points
CNV	37,205	242
DME	11,348	242
Drusen	8616	242
Normal	26,315	242
Total	83,484	968

### ResNet

ResNets—developed by He et al. [13], a Microsoft team—feature a higher number of layers than previous networks. The deepening of neural networks is crucial to improving network performance; however, deepening the number of network layers in the learning stage of deep learning often hinders the learning process and hence undermines performance. By contrast, the incorporation of an identity mapping structure into a ResNet creates a direct proportion between the number of layers and the level of performance. Table 2 provides the levels of a ResNet’s six hyperparameters: number of kernel (*X*_1_), kernel size (*X*_2_), pooling size (*X*_3_), layer (*X*_4_), activation function (*X*_5_), and optimizer (*X*_6_).

**Table 2:** Levels of a ResNet’s hyperparameters

Hyperparameter	Level			
	1	2	3	4
Number of Kernel (*X*_1_)	8	16		
Kernel size (*X*_2_)	3	5		
Pooling size (*X*_3_)	4	8	16	32
Layer (*X*_4_)	14	20		
Activation function (*X*_5_)	Tanh	SELU	Leaky ReLU	ReLU
Optimizer (*X*_6_)	SGD	Adam		

Note that the learning rate (η) and a very small constant (ε) of optimizer Adam in Table 2 were set up to 0.001 and e^−8^ respectively. In addition, a constant was equal to 1.050 and a regulated parameter was set up as 1.6732 for activation function SELU. A gradient parameter (*λ*) was set up as 0.03 for activation function Leaky ReLU.

### Uniform design

Scientific research usually involves multiple experiments, which can be costly and time-consuming. Uniform design improves experimental quality and reduces production time and cost. Uniform design is a multifactor and multilevel experimental design method that features uniform scattering of design points, which enhances the representativeness of each experimental point [8–10, 14]. In this design, a suitable uniform layout is determined for experiments, and a regression analysis is used to obtain the optimal parameters and solution.

A uniform layout is denoted using *U*_*n*_(*n*^*s*^), where *U* refers to uniform layout, *n* is the number of levels (equal to the number of experiments), and *s* is the number of hyperparameters. A uniform layout has (*n* − 1) rows if *n* is a prime number. An even-number uniform layout is constructed by first establishing an odd-number uniform layout and then removing the last row of the layout. Table 3 describes the *U*_12_(12^6^) uniform layout established in this study.

**Table 3:** *U*_12_(12^6^) uniform experiment layout

Number of experiences	Hyperparameter					
	*X*_1_	*X*_2_	*X*_3_	*X*_4_	*X*_5_	*X*_6_
1	8	5	8	20	Tanh	Adam
2	16	5	32	14	Tanh	SGD
3	8	5	4	14	Tanh	Adam
4	16	5	16	20	SELU	SGD
5	8	5	32	14	SELU	SGD
6	16	5	8	14	SELU	Adam
7	8	3	16	20	Leaky ReLU	SGD
8	16	3	4	20	Leaky ReLU	Adam
9	8	3	8	14	Leaky ReLU	Adam
10	16	3	32	20	ReLU	SGD
11	8	3	4	20	ReLU	Adam
12	16	3	16	14	ReLU	SGD

### Signal-to-noise ratio and model assessment

To maximize the AI model’s accuracy and minimize its standard deviation, signal-to-noise ratio (SNR) was adopted as an indicator of measurement quality. An SNR for robust design is obtained only by repeating each hyperparameter combination three or more times, with a high SNR indicating high quality. Equation (1) was used to calculate an SNR with consideration of variance and the difference between predicted and actual values.

1 $${\text{SNR}}_{i} = - 10\log \left[ {\left( {\overline{t}_{i} - m} \right)^{2} + \sigma_{i}^{2} } \right],$$

where ${\text{SNR}}_{i}$ is the SNR of *i*th hyperparameter combination; $\overline{t}_{i}$ and $\sigma_{i}$ are respectively the mean and standard deviation of accuracy for the *i*th hyperparameter combination; and *m* is the targeted accuracy (*m* = 1), $i = 1, 2, \ldots , 12$.

2 $$\overline{t}_{i} = \frac{{\mathop \sum \nolimits_{j = 1}^{p} t_{ij} }}{p}$$

and

3 $$\sigma_{i}^{2} = \frac{{\mathop \sum \nolimits_{j = 1}^{p} \left( {t_{ij} - \overline{t}_{i} } \right)}}{p - 1},$$

where $t_{ij}$ is the *j*th accuracy testing of the *i*th hyperparameter combination, and $p = 1, 2, 3$. Therefore, the optimal objective is to maximum Eq. (1). A confusion matrix can be used to calculate indicators such as accuracy, sensitivity, and false negative rate in assessing model performance [15]. Therefore, this study employed a confusion matrix for model assessment (Table 4). As depicted in (4), ACC is the overall accuracy of the model; high ACC indicates good model performance.

4 $${\text{ACC}} = \frac{{\mathop \sum \nolimits_{i = 1}^{q} T_{ii} }}{{\mathop \sum \nolimits_{i = 1}^{q} \mathop \sum \nolimits_{j = 1}^{q} T_{ij} }}\quad (q = 1,2,3,4)$$

**Table 4:** Confusion matrix

Actual values	Predicted values			
	Category 1	Category 2	Category 3	Category 4
Category 1	*T*_11_	*T*_12_	*T*_13_	*T*_14_
Category 2	*T*_21_	*T*_22_	*T*_23_	*T*_24_
Category 3	*T*_31_	*T*_32_	*T*_33_	*T*_34_
Category 4	*T*_41_	*T*_42_	*T*_43_	*T*_44_

## Results and discussion

In the *U*_12_(12^6^) uniform experiment layout (Table 3), each hyperparameter combination was used for three rounds of random data grouping to construct three ResNet models; the mean and standard deviation of accuracy of each were obtained and the SNR calculated using (1). Subsequently, the performance of all hyperparameter combinations listed in Table 3 was assessed to determine the optimal combination. Table 5 presents the uniform experiment result based on the training data. Table 6 provides the SNR result of the uniform experiment based on the training and validation data. To achieve an accurate, stable model, the SNRs of the training and validation data were summed for each hyperparameter combination to identify the combination with the highest accuracy and stability. Combination 4 had the optimal hyperparameter combination, with an SNR of 31.87 for the training data and 24.31 for the validation data for a total of 56.19, the largest sum among all combinations (Table 6).

**Table 5:** Accuracy of uniform experimental results obtained using training data

Hyperparameter combinations	Results					
	Accuracy in the 1st experiment	Accuracy in the 2nd experiment	Accuracy in the 3rd experiment	Mean	Standard deviation	SNR
1	0.9340	0.9338	0.9378	0.9352	0.002254	23.76
2	0.9586	0.9463	0.9602	0.9550	0.007606	26.81
3	0.9388	0.9365	0.9398	0.9383	0.001692	24.20
4	0.9678	0.9780	0.9805	0.9754	0.006728	31.87
5	0.9567	0.9551	0.9541	0.9553	0.001312	26.99
6	0.9404	0.9398	0.9418	0.9406	0.001026	24.53
7	0.9557	0.9557	0.9558	0.9557	0.000058	27.07
8	0.9487	0.9544	0.9449	0.9493	0.004782	25.86
9	0.9408	0.9406	0.9408	0.9407	0.000116	24.54
10	0.9528	0.9722	0.9710	0.9653	0.010871	28.79
11	0.9536	0.9527	0.9556	0.9539	0.001484	26.73
12	0.9725	0.9761	0.9662	0.9716	0.005011	30.80

**Table 6:** SNRs of the uniform experiments

Hyperparameter combinations	Results		
	SNRs obtained using training data	SNR obtained using validation data	Summed SNR
1	23.76	18.48	42.24
2	26.81	24.01	50.83
3	24.20	18.35	42.56
4	31.87	24.31	56.19
5	26.99	22.30	49.30
6	24.53	22.39	46.93
7	27.07	23.96	51.04
8	25.86	22.74	48.62
9	24.54	22.06	46.61
10	28.79	23.68	52.48
11	26.73	24.39	51.13
12	30.80	23.42	54.22

Hyperparameter combination 4 was tested thrice using the 968 test data points specified in Table 1. Table 7 lists the test results. The mean accuracy was 0.9848 and the mean false negative rate 0.0150. The results verified the excellent performance of the hyperparameter combination obtained using the proposed method.

**Table 7:** Test results for hyperparameter combination 4 (test data)

Experiment number	Results	
	Accuracy	False negative rate
1	0.9907	0.0092
2	0.9824	0.0175
3	0.9814	0.0185
Mean	0.9848	0.0150

This study subsequently compared a ResNet model with the CNN model proposed by Najeeb et al. [11]. The CNN model had 32 kernels, a kernel size of 3, and a stride of 1 in the convolutional layer; a pooling size of 2 in the pooling layer; a 20% dropout; a ReLU activation function; and two fully connected layers, with one comprising 256 neurons and the other comprising 4. Najeeb et al. [11], who employed the same dataset used in the present study to test their CNN model, obtained an accuracy of 0.9566 and a false negative rate of 0.043.

Ultimately, the present study’s ResNet model compared with an AlexNet model [16]. Refer to [16], there are 4096, 4096 and 4 neurons for three fully connected layers respectively, and extra 50% dropout was added for the first two fully connected layers and all activation functions were set up as ReLU, and optimizers were SGD. Three experiments of the AlexNet model with the same test dataset resulted a mean accuracy of 0.9803 and a mean false negative rate of 0.0188.

Table 8 summarizes the evaluation outcomes for the present study’s model, CNN model of Ref. [11] and AlexNet model of Ref. [16]. In comparison with the CNN model and AlexNet model, the present study’s ResNet model had superior performance, with a mean accuracy of 0.9848 and a mean false negative rate of 0.0150.

**Table 8:** The experimental results of tree neural network models (test data)

Model	Result			
	Best accuracy	Mean accuracy	Standard deviation	Mean false negative rate
ResNet	0.9907	0.9848	0.0051	0.0150
AlexNet	0.9876	0.9803	0.0063	0.0188
CNN	0.9566	–	–	0.0430

## Conclusions

The ResNet model established using uniform design exhibited high stability, which is attributable to the study’s strict focus on achieving both high accuracy and low standard deviation in the model establishment process. Uniform design was used to optimize the ResNet model’s hyperparameters because this design method—with its uniform distribution of experimental points—facilitates effective identification of the representative hyperparameters, reduces the time required for hyperparameter design, and fulfils the requirements of a systematic hyperparameter design process.

To verify the performance of the proposed method in finding the optimal hyperparameter combination, an open dataset was divided into training, validation, and test datasets for the testing of hyperparameter combinations. The test results were then compared with those obtained using the CNN model developed by Najeeb et al. [11]. According to the comparison, the present study’s ResNet model, with its optimal hyperparameter combination determined using the proposed method, outperformed the CNN model of Najeeb et al. [11] in both accuracy and false negative rate, which verified the practicality of the proposed method.

## Data Availability

The datasets analysed during the current study are available in the Large Dataset of Labeled Optical Coherence Tomography (OCT) and Chest X-Ray Images 2018, https://data.mendeley.com/datasets/rscbjbr9sj/3.

## Abbreviations

CNV: Choroidal neovascularization
DME: Diabetic macular edeman
OCT: Optical coherence tomography
AI: Artificial intelligence
CNN: Convolutional neural network
ResNet: Residual neural network
SNR: Signal-to-noise ratio
ACC: Overall accuracy of the model
